# New Therapeutic Targets for Intraocular Pressure Lowering

**DOI:** 10.1155/2013/261386

**Published:** 2013-07-16

**Authors:** A. Rocha-Sousa, J. Rodrigues-Araújo, Petra Gouveia, João Barbosa-Breda, S. Azevedo-Pinto, P. Pereira-Silva, A. Leite-Moreira

**Affiliations:** ^1^Department of Sense Organs, Faculty of Medicine, University of Porto, 4200-319 Porto, Portugal; ^2^Department of Ophthalmology, São João Hospital, 4200-319 Porto, Portugal; ^3^Department of Physiology and Cardiothoracic Surgery, Faculty of Medicine, University of Porto, 4200-319 Porto, Portugal

## Abstract

Primary open-angle glaucoma (POAG) is a leading cause of irreversible and preventable blindness and ocular hypertension is the strongest known risk factor. With current classes of drugs, management of the disease focuses on lowering intraocular pressure (IOP). Despite of their use to modify the course of the disease, none of the current medications for POAG is able to reduce the IOP by more than 25%–30%. Also, some glaucoma patients show disease progression despite of the therapeutics. This paper examines the new described physiological targets for reducing the IOP. The main cause of elevated IOP in POAG is thought to be an increased outflow resistance via the pressure-dependent trabecular outflow system, so there is a crescent interest in increasing trabecular meshwork outflow by extracellular matrix remodeling and/or by modulation of contractility/TM cytoskeleton disruption. Modulation of new agents that act mainly on trabecular meshwork outflow may be the future hypotensive treatment for glaucoma patients. There are also other agents in which modulation may decrease aqueous humour production or increase uveoscleral outflow by different mechanisms from those drugs available for glaucoma treatment. Recently, a role for the ghrelin-GHSR system in the pathophysiology modulation of the anterior segment, particularly regarding glaucoma, has been proposed.

## 1. Introduction

Glaucoma is a progressive optic neuropathy caused by death of the retinal ganglion cells (RGCs) and is the leading cause of irreversible blindness worldwide. The mechanism by which this progressive RGC death occurs is not fully understood. It is clear that multiple causes may give rise to the common effect of ganglion cell death. 

Clinically, it is well accepted that the major risk factor for glaucoma is elevated intraocular pressure (IOP) [1, 2]. In open angle glaucoma (OAG), elevated IOP occurs from an imbalance between production and outflow of aqueous humor (AH).

The mechanical theory argues the importance of direct compression of the axonal fibers and support structures of the anterior optic nerve by elevated IOP resulting in the death of the RGCs. Lowering the IOP (baroprotection) remains the only current therapeutic approach for preserving visual function in glaucoma patients. The six classes of drugs available for glaucoma treatment (miotics, beta-blockers, alfa-agonists, epinephrine derivatives, carbonic anhydrase inhibitors, and prostaglandin analogues) act by decreasing aqueous humor production and/or by improving trabecular meshwork-Schlemm's canal or uveoscleral outflow.

Better knowledge about cellular and molecular glaucomatous changes in the aqueous production and outflow pathways opened a new horizon for new hypotensive class agents. The main cause of elevated IOP in primary open angle glaucoma (POAG) is thought to be an increased outflow resistance via the pressure-dependent trabecular outflow system by an increased accumulation of extracellular matrix (ECM) material in the trabecular meshwork, due to a disturbed balance between ECM deposition and degradation. 

From all of the glaucoma medications, approved for clinical use, that decrease the intraocular pressure, only analogues prostaglandin (PG) may have a role on modulation of the molecular changes that occurred in glaucoma patients. Some studies showed that PG's analogues may induce stimulation of collagenases and other matrix metalloproteinases [3, 4] which is thought to result in dilated spaces between ciliary muscle bundles. However, other studies, using both light and electron microscopy, have found no evidence of dilated spaces between ciliary muscle bundles or other alterations in the ciliary muscle or other ocular tissues in monkeys treated with PGF2a [5]. 

There are 2 main new therapeutic approaches to increase outflow facility in TM. The first one includes the alteration in activities or behavior of TM cells [6]. Some agents affect cell volume and shape and loosen cell-to-cell junction and/or cell-to-extracellular matrix adhesion within the TM and inner wall of Schlemm's canal. Considerable evidence has shown that TM cells are highly contractile and play an active role in aqueous humor dynamics. Modulation of contractility of TM represents the second possible therapeutic concept within the TM. It has been shown that TM tissues possess smooth muscle cell-like properties. The contractile and relaxation properties of TM cells are regulated by several enzymes, which have become experimental therapeutic targets for lowering IOP [7–12].

There are also new therapeutic approaches to decrease aqueous humour production or to improve increased uveoscleral outflow by different subcellular pathways from those that already exist [13–16].

Moreover, a significant number of patients presenting with glaucoma continue to lose vision despite responding well to therapies that lower eye pressure. “Non-IOP-dependent” risk factors appear to be responsible for approximately 30–70% of glaucoma cases [17–20]. Enhancement of optic nerve blood supply and neuroprotection are potential treatment strategies for glaucoma, but are beyond the scope of this paper.

**Figure 1:**
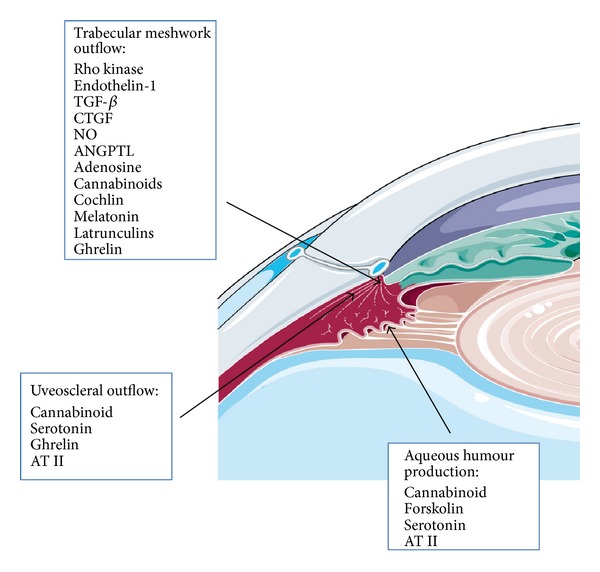
New therapeutic targets that lower intraocular pressure. Mechanisms of action are indicated in the figure.

In this paper, we discuss potential concepts for the future treatment of OAG based on hypotensive effect by mechanisms of action that are different from current therapeutic approach that is available for clinical use (see Figure 1 and Table 1). 

## 2. Materials and Methods

The focus of this paper is new therapeutics targets for IOP lowering. Excluded compounds are those with no proved intraocular hypotensive effect and those that act by same mechanism of current therapeutic approach. A Systematic revision of studies published between 2000 and 2013 in English, Spanish and Portuguese in MEDLINE, EMBASE, and Scopus was done. Search words used included *glaucoma therapy, intraocular pressure, trabecular meshwork, baroprotection, novel drugs, and open angle glaucoma.*

## 3. Results and Discussion

### 3.1. Rho Kinase

Originally identified as a downstream effector of the small GTPase Rho [21], Rho-associated kinase, also known as ROCK, belongs to the AGC (PKA/PKG/PKC) family of serine/threonine kinases [22].

There are two Rho-kinase isoforms, ubiquitously expressed as Rho-kinase *α*/ROCK2/ROK*α* and Rho-kinase *β*/ROCK1/ROK*β*/p160ROCK, collectively referred to as Rho-kinase. They are highly homologous in their molecular structure (65% of the same amino acid sequence and 92% homology in kinase domains) and also in their function. Knockout null homozygous mice developed a similar phenotype between the two isoforms (eyes open at birth and omphalocele), demonstrating that the biological functions of both isoforms are redundant and cannot be separated. Also, they both act on the same major downstream substrates [22]. The Rho signaling pathway, mediated through ROCK, plays a major role in regulation of smooth muscle contraction, through the regulation of the actin-myosin filament bundles [16, 23]. Activated RhoA/ROCK leads to the phosphorylation of myosin light chain (MLC) [24], in which combined with actin induces a calcium-independent smooth muscle contraction, with formation of actin stress fibers and focal adhesions, allowing cytoskeletal rearrangement, cell motility, and proliferation [25].

Expression of RhoA GTPase, ROCKs (both ROCK 1 and ROCK2), and MLC in TM and ciliary muscle (CM) cells from humans and other species has been confirmed [16]. Physiological agonists that activate Rho kinase signaling pathway, such as endothelin-1, transforming growth factor (TGF), thrombin, and lysophospholipids, reduce aqueous humor outflow in animals [26]. Inhibition of ROCK was demonstrated to contribute to effects such as smooth muscle relaxation and alteration of intercellular junctions in the TM of the eye [27], by decreasing MLC phosphorylation [28]. Inhibitors of ROCK and Rho GTPase lower IOP in both animal models [3] and humans; a phase II clinical trial showed that K-115 has the ability to lower intraocular pressure in humans with POAG or OH [29].

Apart from this, Rho GTPase and ROCK inhibitors have potential neuroprotective effects by increasing ocular blood flow and enhancing survival of retinal ganglionary cells and axon regeneration in animal models [30]. The first Rho-kinase inhibitor was Y-27632. It is not specific for ROCK (neither for any of its isoforms) since it affects other protein kinases in higher concentrations, such as PKA and PKC. Fasudil (HA-1077) is also not specific and is the only clinically approved ROCK inhibitor so far, being used for cerebral vasospasm after subarachnoid hemorrhage in Japan [21, 22]. 

During the analysis of ROCK inhibitors as a potential glaucoma topical pharmacological treatment some problems arose. First of all, the lack of specificity of the inhibitors which in high concentrations also inhibit other kinases. Also, little tolerability was described, since ROCK inhibitors result in vasodilation, resulting in ocular hyperemia, which is transient, but nonetheless the main ocular side effect described so far. Regarding these last two problems, one possible solution for many authors is the combination with other IOP lowering drugs, allowing for the use of low ROCK inhibitor concentrations. Another possible solution might be the use of a prodrug, converted into a more active compound once it passes through the cornea and is present in the anterior chamber (ATS907 is an example and is currently in phase II clinical trials) [21, 22].

In conclusion, ROCK inhibitors have the ability to lower IOP in patients with POAG and/or ocular hypertension (OH) by increasing the outflow through the trabecular meshwork.

### 3.2. Endothelin-1

 Endothelin-1 (ET-1) is a vasoconstrictive peptide that originates in the endothelium [31, 32]. Elevated concentration of endothelin-1 has been documented in the aqueous humor of patients with glaucoma. A highly significant correlation has been found between IOP and ET-1. However, there is yet to be proven a causative relation [33].

Previous studies have stated that ET-1 is capable of inducing the contraction of both trabecular meshwork cells and the cellular matrix [31, 32]. The contraction seems to be triggered by activation of the RhoA/ROCK pathway [24] which leads to increase in the outflow resistance and consequently to an IOP elevation [34, 35]. An ETA receptor antagonist would be ideal to lower IOP. Some experiments, however, suggest a correlation between ET-1 and NO: ET-1 is capable to activate NOS isoform III [34] which will induce TM relaxation if high levels of NO are present [36]. 

ETA receptor antagonists constitute a potential new treatment modality to manage glaucoma through IOP reduction. ETB receptor antagonists also seem to play a neuroprotective role by impeding RGCs apoptosis.

### 3.3. Transforming Growth Factor-*β*

The transforming growth factor-*β* (TGF-*β*) superfamily is a group of structurally related multifunctional regulatory proteins. There are at least three isoforms: TGF-*β*1, TGF-*β*2, and TGF-*β*3 [37]. In normal physiology, cell processes influenced by TGF-*β* are proliferation, recognition, differentiation, and apoptosis [38]. TGF-*β* was found to be elevated in the AH of normal [39] as well as glaucomatous human eyes, but in higher levels in the latter [40], more specifically, higher levels of TGF-*β*2 in POAG, being the other isoforms elevated in other forms of glaucoma [38]. Additionally, studies showed that TGF-*β*2 is produced mainly in the eye, with minimum serum concentration in patients with high ocular levels and no correlation between AH and serum levels [41]. Several studies proved TGF-*β* to be a cause of elevated IOP. A TGF-*β*2 infusion in an animal's anterior eye segment perfusion culture significantly increased IOP [38]. Additionally, Shepard et al. [42] observed an increased IOP and reduced AH outflow in rodents following adenoviral gene transfer of active human TGF-*β*2. We can then assume that elevated levels of TGF-*β*2 in the AH lead to an increased risk of developing elevated IOP, and consequently placing the patients at risk for optic nerve damage and vision loss.

Trivedi et al. postulate that the increased risk for developing glaucoma with increasing age might be related to the rise of TGF-*β*2 [43].

As for the changes that take place in the TM, TGF-*β*2 leads to a decrease in its cellularity, by inhibition of proliferation, stimulation of apoptosis, and phagocytosis. At the same time, it changes the number of cells, also cell phenotype is affected, shifting to a secretory type, leading to an increase in the collagen content of the ECM, which may lead to TM obstruction and decrease of AH outflow [44].

TGF-*β*1 and TGF-*β*2 not only have shown to increase ECM production, also inhibit its degradation; both isoforms *β*1 and *β*2 lead to overexpression of genes encoding ECM proteins hence increasing its production [38]. Gottanka et al. showed how *in vitro* treatment with TGF-*β*2 led to diminished outflow facility, thanks to the accumulation of fibrillar matrix materials under the inner wall of the canal of Schlemm, and also by diminished length of the canal [39]. Apart from acting directly, TGF-*β*2 isomer also induces expression of other effectors, such as connective tissue growth factor, thrombospondin-1, fibronectin, cochlin [45], collagen types IV and VI, and plasminogen activator inhibitor-1 (PAI-1), both in cultured human and animal TM cells [38]. PAI-1 increased levels result in decreased activity of MMPs, since it acts as a potent inhibitor of the plasminogen/plasmin system required to activate MMPs. This is another pathway through which TGF-*β*2 increases ECM in the TM of glaucomatous eyes [46]. 

Thrombospondin-1 is expressed intensely throughout the TM in glaucomatous eyes; it acts as a potent activator of TGF-*β* and also has its levels increased by it, making it a self-amplifying mechanism for TGF-*β*2 [47]. 

Several molecules exist to counteract or terminate TGF-*β*'s effects in the eye. Bone morphogenetic proteins 4 and 7 (BMP-4 and BMP-7, resp.), members of the TGF-*β* superfamily, prevent ECM deposition and antagonize the fibrogenic actions of TGF-*β*2 on human TM cells, respectively [38]. In addition to this, there are molecules that bind to BMPs and inhibit them hence releasing TGF-*β*'s effects. Some of these BMP-binding molecules, such as follistatin, gremlin, and chordin, are expressed in TM cells, and higher levels of gremlin have been found in TM cells from POAG eyes compared with nonglaucomatous eyes [45]. Treatment of TM cells with recombinant gremlin induces expression of typical TGF-*β* target genes, such as those for fibronectin, collagen type I, elastin, or PAI-1 [48]. Although TGF-*β*1 is not the main isoform expressed in POAG, we will devote the next paragraphs to its function. The transfer of active TGF-*β*1 gene into rat eyes led to anatomical changes in the anterior segment that resulted in increased IOP [38]. TGF-*β*1 is also proven to induce a myofibroblast-like phenotype in human TM cells *in vitro* hence leading to an increase in contractility of TM cells and decrease in outflow facility [49]. Formation of actin stress fibres in TM cells triggered by TGF-*β*1 is mediated by protein kinase C and Rho GTPase [50]. Both TGF-*β*1 and TGF-*β*2 increase fibronectin, but also tissue transglutaminase, in turn leads to an irreversible cross-linking of fibronectin [51]. *In vitro*, fibronectin enhances TM cell-mediated contraction, thus, facilitating the connection of TM cells to the surrounding matrix and the formation of stress fibres [38]. Also, the lysyl oxidase family of proteins (LOX, LOXL 1–4) has the ability to cross-link ECM proteins turning them insoluble and stiffening the ECM, and their expression is induced by treatment with all three isoforms of TGF-*β* [45]. There is still no report of dysregulated LOX expression in POAG. Moreover, TGF-*β*1 increases connective tissue growth factor and elastin production in human TM cells *in vitro*, which could contribute to outflow resistance [39]. Also TM cell myocilin was increased following TGF-*β*1 exposure *in vitro* [52]. Myocilin also contributes to TM outflow resistance and elevation of IOP by interacting with the TM. Additionally, accumulation of myocilin in the TM occurs in glaucomatous eyes [53].

As shown above, TGF-*β* plays a central role in the activity of several of the physiological pathways leading to elevated IOP in glaucomatous eyes. Evidence supports the fact that the main target of TGF-*β* in POAG involves the TM. TGF-*β*1 and TGF-*β*2 have been identified as potential modulators of aqueous outflow facility through ECM remodeling and also TM cellularity and contraction, making these proteins a possible target for glaucoma treatment [38].

### 3.4. Connective Tissue Growth Factor

Connective tissue growth factor (CTGF) is a four-module protein that performs as a signal mediator in pathways activated by TGF-*β* which on the other hand increases CTGF levels [54, 55]. Different types of glaucoma express different types of TGF in the aqueous humor: TGF-*β*2 is highest in the aqueous humor of POAG patients [41, 56] while patients with PXG present increased expression of TGF-*β*1 [49]. Previous studies have documented an evident effect on ECM production on cultured Tenon's capsule of PXG patients with elevated TGF-*β*1, while cultures from POAG patients, with high TGF-*β*2, experienced cell migration and collagen contraction [57]. As far as CTGF is concerned, its levels were higher in the aqueous humor of PXG patients and the magnitude of elevation seemed to be related to the severity of the disease [58].

Studying the mechanism through which CTGF contributes to the development and progression of PXG has proven to be difficult mostly due to the ability of this protein to bind receptors that activate divergent signaling pathways such as p42/44 MAPK [59], PI3J [60], RhoGTPase [61], and p38 MAPK [62]. These pathways are linked and authors have previously suggested the importance of these links in understanding the CTGF-controlled gene transcription [63]. In POAG, outflow resistance at the juxtacanalicular regions causes aqueous humor outflow resistance and increases IOP [64, 65]. The quantity and quality of TM extracellular matrix have been suggested as two of the contributing factors as POAG demonstrates increased ECM at the juxtacanalicular region outflow [66, 67]. Increased expression of CTGF induces ECM protein expression especially fibrillar and basement membrane collagens [68]. In fact, CTGF appears to directly correlate with accumulation and deposition of collagen I [69]. Browne et al. showed that CTGF increased expression of fibrillin-1 [63], corroborating previous studies that had elucidated the role of high levels of fibrillin or abnormal aggregation of fibrillin-containing microfibrils in the pathogenesis of PX syndrome [70]. In fact, in PX syndrome, there are higher levels of fibrillin containing fibrils in the extracellular matrix and fibrillin is one of the components present in the elastic fibrils in PX material. 

Some studies have emphasized the role of TGF-*β*2 in enhancing expression and deposition of ECM molecules such as fibronectin and collagens IV and VI, which are all components of the fibrillar ECM in the JCT [51, 71, 72]. Furthermore, these experiments also revealed decreased activity of matrix metalloproteinases (MMPs), which normally degrade ECM compounds [46]. Recent study demonstrated high IOP and optic nerve damage in mice eyes in response to overexpressed CTGF [73]. This action was counteracted by Rho kinase inhibitors. The authors also confirmed that CTGF induces fibronectin as well as actin stress fibers and contractility in trabecular meshwork cells, consequently elevating IOP. Iyer and colleagues corroborated these findings by stating that Rho GTPase and Rho kinase regulate CTGF expression in TM cells, possibly through controlling actomyosin-based contraction and the levels of free G-actin [74]. Also, the changes in the actomyosin organization and myosin II activity induced by CTGF were associated with higher levels of fibronectin and laminin in TM cells. Neuromedin U is a neuropeptide which is induced by CTGF in TM cells. It facilitates AH outflow through activating actomyosin contraction of TM cells [74]. 

These data suggest the existence of an interaction between Rho/Rho kinase pathway activity, CTGF expression, and the contractile properties of TM cells. These interactions regulate the homeostasis of ECM synthesis and AH drainage through the trabecular pathway. Interfering with CTGF expression will therefore have effects on the ECM synthesis and AH outflow and might prove beneficial in the treatment of glaucoma.

### 3.5. Nitric Oxide

The maxi-K channel is a calcium-dependent potassium channel with a dense distribution in the trabecular meshwork [75, 76]. Its function is to regulate smooth muscle tone and, in fact, it acts as target for several mediating factors whose ultimate goal is smooth muscle relaxation [36]. 

NO is produced from L-arginine by NOS in the trabecular meshwork and cellular matrix [77]. It binds the maxi-K channel and triggers increased expression of cGMP [36, 78]. The cGMP-dependent kinase is then activated and phosphorylates the channel, prolonging the amount of time it remains open [79]. As a consequence, the efflux of potassium is greater and culminates in repolarization. At the same time, voltage-dependent calcium L-type channels close and muscle relaxes [80]. NO agonists induce relaxation of trabecular meshwork cells [77] and they appear to do so through changing the conformation of cytoskeletal proteins such as actin, myosin, and tubulin [81]. 

### 3.6. Angiopoietin-Like Molecules

Angiopoietin-like (ANGPTL) molecules are a family of glycoproteins which resemble angiopoietins structurally [82] but fail to bind the angiopoietin receptors Tie1 and Tie2 [83–85]. Despite these, some of the members in this family are potent regulators of angiogenesis [86, 87] and function as mediators in the induction of inflammation and regulation of lipid and glucose metabolism [88–90]. 

Angiopoietin-like 7, previously named cornea-derived transcript 6 (CDT6) [91], is expressed in the human trabecular meshwork and may be induced by treatment with dexamethasone [92–94] as well as by TGF-*β*2 [95], an overexpressed growth factor in the aqueous humor of glaucoma patients [41]. A beagle model of POAG demonstrated ANGPTL7 elevation in the dogs' aqueous humor which was consistent with the elevation of the same protein in the aqueous humor of glaucoma patients. Using the same animal model, the authors detected increased ANGPTL7 with disease progression. Increased expression of ANGPTL-7 is thought to arise due to elevated IOP [95]. 

The gene for ANGPTL-7 is located near TCF/LEF transcription factors which are key for the activation of the WNT/b-catenin signaling pathway [96]. This pathway has been previously linked with elevated IOP and glaucoma [97, 98]. The WNT secreted glycoproteins transmit signals by binding to frizzled transmembrane receptors. This results in dephosphorylation and consequent nuclear translocation of b-catenin. B-catenin will then activate expression of genes through interaction with transcription factors of the TCF/LEF family. It is therefore possible that the activation of ANGPTL7 could be mediated by the activation of the WNT pathway regulatory elements [98].

It has been studied on how ANGPTL-7 influences and modifies the trabecular meshwork. Peek et al. have postulated that ANGPTL-7 increases expression of proteoglycans and collagens types I and V [99]. Gabelt and Kaufmanwent further, stating that changes in collagen could alter the trabecular meshwork in a way compatible with increased resistance and consequent decreased aqueous humor outflow and, henceforth, cause ocular hypertension [100]. The increased expression of collagen in the sclera could also limit the aqueous humor outflow through the uveal-scleral pathway which would result in elevated IOP [101]. Studies in transgenic mice have shown that collagen I overexpression was associated with increased IOP and optic nerve damage, as observed in human POAG [102, 103]. Comes et al., on the other hand, verified decreased expression of collagen IA1, fibronectin collagen type VA1, versican, and to a lesser extent myocilin. Furthermore, induction of MMP1 was noted [104]. When ANGPTL-7 was silenced, the reverse protein expression occurred. The authors speculate that the different results concerning collagen I may be due to different cells used (transformed versus primary trabecular meshwork cells) or due to a posttranscription regulation of collagen I in these cells. Previous studies have revealed how fibronectin may be implicated in increasing outflow resistance [105]. Since overexpression of ANGPTL-7 reduces levels of fibronectin, Comes and colleagues suggest that this protein may actually facilitate rather that restrict aqueous humor outflow. This effect is supported by the increased expression of MMP1, a collagenase which has been linked to increased outflow in organ cultures [106]. 

Further studies are needed in order to confirm the benefit of a ANGPTL-7 agonist in the IOP lowering.

### 3.7. Adenosine

Adenosine modulates several physiological and pathophysiological pathways through its G protein-coupled receptors [107], and increased levels are found in retinal ischemia and elevated IOP [16]. There are four adenosine receptor subtypes known so far (A1, A2a, A2b, and A3), and A1, A2a, and A3 agonists and A3 antagonists have been considered potential IOP lowering drugs. The mechanism proposed is the secretion of MMPs in the TM, which lead to remodeling of the ECM and consequently an increase in TM outflow [16]. As for A1 agonists, preclinical studies with topical treatment showed statistically significant IOP decrease in primates [108], rabbits [109], and mice [14]. A2a agonists are currently in clinical trial phase with the expectation of lowering IOP, but interestingly, Avila et al. (2001) stated that agonists for these receptors given to monkeys increased IOP [14]. Regarding A3 receptor subtype, agonists are under clinical trial, since an unexpected finding in a previous study showed a decrease in IOP [110], but also antagonists are proposed to lower IOP, since the activation of A3 receptors leads to the activation of chloride channels in human nonpigmented ciliary epithelial cells, which potentially may lead to the increase in AH production [107, 111].

### 3.8. Latrunculins

Latrunculins, macrolides discovered in marine sponges, have the ability to inhibit actin polymerization [29], disrupting the TM's cytoskeleton and consequently its integrity, which results in increased outflow [112]. This increase in outflow may result from expansion of the space between SC and the TM's collagen beams and/or by increased openings between SC's inner wall cells [107]. Latrunculin-A and latrunculin-B intracamerally or topically induce a high increase in the TM's outflow and reduce IOP in monkeys [5]. Clinical trials have been undertaken, but so far no significant results were found. Several side effects were described with the highest doses used (0,02% and 0,05%), namely, mild ocular redness, irritation, and transient increase in central corneal thickness of <2,5% [113].

### 3.9. Cochlin

Cochlin is the product of the coagulation factor C homology gene (COCH) and is an ECM protein of unknown function. Evidence suggests association between cochlin and glaucomatous trabecular meshwork tissue [114] and that cochlin acts by cellular mechanosensitive mechanism [115–117]. 

Cochlin was identified in glaucomatous but not normal human TM [114]. Early cochlin protein expression was found in the TM of the glaucomatous DBA/2J mouse model as early as 3 weeks and the level of cochlin reaches a plateau around 6–8 months of age when elevation in IOP is observed [118]. 

Stretch activated channels (SACs), such as TREK-1, function as mechanotransducers involved in pressure regulation [115, 116], and cochlin expression has been previously shown to result in coexpression of TREK-1 [119]. Recent studies show that cochlin is involved in regulation of intraocular pressure in DBA/2J potentially through mechanosensing of the shear stress by demonstrating that overexpression and downregulation of cochlin increase and decrease intraocular pressure (IOP), respectively. Reintroduction of cochlin in cochlin-null mice increases IOP; injection of exogenous cochlin also increased IOP and increasing perfusion rates increased cochlin multimerization, which reduced the rate of cochlin proteolysis by trypsin, and proteinase K. The cochlin multimerization in response to shear stress suggests its potential mechanosensing [117].

### 3.10. Cannabinoids

There are two different receptor subtypes for cannabinoids, CB1 and CB2, and several compounds can activate them. Human endoligands of the cannabinoid receptor system are arachidonic acid-like substances and are called endocannabinoids. Although the expression of CB1 and CB2 has been described in ocular tissues, the main cannabinoid receptors expressed in the eye are CB1. The distribution of these receptors in the human eye has been studied, with high presence in corneal epithelium and endothelium, trabecular mesh, nonpigmented ciliary epithelium, ciliary muscle, and photoreceptor outer segments, with lesser intense presence in several other eye structures [120, 121]. Since CB1 receptor subtype is present in both structures where AH is produced and removed, it might induce an IOP reduction through a decrease in humor production and also an increase in its outflow, both through the TM and the uveoscleral routes [122]. Cannabinoids can also produce vasodilation of anterior uvea efferent blood vessels, hence, improving aqueous outflow [123]. Currently, the main hypotensive mechanism is considered to be increased outflow, by producing an increase in the dimensions of Schlemm's canal [124]. Noladin ether (endocannabinoid agonist) can induce MMP activation, which leads to remodeling of the TM and also reduces the production of actin stress fibers and focal adhesions [125]. The ocular hypotensive properties of cannabinoids are dependent on the *β*-adrenergic receptors stimulation. The cannabinoids reduce IOP by acting as indirect sympatholytics [126]. Cannabinoids can induce COX-2 expression, resulting in higher levels of PGE2 and MMPs, which modulates aqueous outflow resistance [127].

Marijuana (*Cannabis sativa*) is known to reduce IOP via systemic exposure; there are reports of IOP decrease from 30 mmHg to 15 mmHg [15]. The topical route was studied using WIN 55212-2, an agonist of CB1 and CB2 receptor subtypes. In normotensive cynomolgus monkeys, topically administered WIN 55212-2 by 0.5%, reduced IOP levels by 19%. Reduced aqueous humor flow was identified, but no changes in outflow facility were noted [123]. Also, the sublingual route was studied. Δ-9-THC (delta-9-tetrahydrocannabinol), which is the main psychoactive element, was able to reduce IOP over placebo, for only 4 hours (23.5 mmHg versus 27.3 mmHg; *P* = 0.026). This effect was not observed with topical application [26]. The inhalatory and intravenous routes have also proven to be successful in the reduction of IOP [122].

There is currently a proof that cannabinoids can lower IOP in humans. However, the development of tolerance and significant systemic toxicity appears to limit the usefulness of this potential treatment, making it an end-line treatment when available.

### 3.11. Melatonin

Melatonin is a neurohormone secreted into the blood mainly from the pineal gland. It is responsible for the regulation of the circadian rhythms of several biological functions and is regulated by the light/dark cycle [16, 128]. Three subtypes of melatonin receptors have been identified, MT1 and MT2 receptor subtypes, negatively coupled with adenylate cyclase, and MT3, coupled with phospholipase C [129].

Changes in melatonin levels in AH in light or dark, associated with IOP changes, suggest a melatonergic regulation of the circadian rhythm of IOP. A study revealed that knock-out mice for MT1 receptor subtype had higher IOP levels during the nocturnal hours than controls or knock-out mice for MT2 receptor subtype at 3 and 12 months of age. Additionally, the administration of exogenous melatonin significantly reduced IOP levels in wild-type mice, but not in the MT1 knock-out mice [130]. 5-MCA-NAT, an MT3 receptor agonist, has shown to reduce IOP by 19% in glaucomatous monkey eyes and by 40% in rabbits. Apparently, part of the mechanism of action is related to the cholinergic and noradrenergic systems, since antagonists of such systems could reverse the effect [131].

### 3.12. Ghrelin

Ghrelin is a 28-amino acid acylated peptide and is the endogenous ligand for the growth hormone secretagogues receptor (GHSR-1a), promoting the release of growth hormone from the pituitary independently from the growth-hormone-releasing hormone receptor [132]. Another variant of ghrelin is its unacylated form, desacyl ghrelin. This variant is identical to ghrelin except for the acyl group in the serine 3, thus, being unable to bind GHSR-1a [133]. 

In recent studies, ghrelin has been proposed to play important roles in the ocular tissue, both in the anterior and posterior segments. Regarding the anterior segment, ghrelin's mRNA was identified in the posterior surface of the iris and in the nonpigmented ciliary epithelium. This peptide was also shown to induce the relaxation of the iris sphincter and dilator muscles [134]. Ghrelin has also been implicated in glaucoma, being its levels significantly decreased in the aqueous humour of patients suffering from POAG and pseudoexfoliation glaucoma when compared to the control group [135, 136]. When comparing ghrelin AH levels from individuals with primary open angle glaucoma and pseudoexfoliation glaucoma, there was no significant difference [136]. The correlation between ghrelin plasma and AH levels remains controversial. 

Ghrelin and desacyl ghrelin effect in the modulation of the intraocular pressure was recently studied in two animal models of osmotically induced intraocular hypertension. Ghrelin promoted a marked and sustained decrease of ocular tension when compared to control, presenting a maximal percentual decrease of 43.8 ± 12.05% in the rabbit and of 33.28 ± 6.47% in the rat. Desacyl ghrelin action did not affect the intraocular hypertension in the rabbit model, thus, indicating a role for GHSR-1a in the hypotensive effect induced by ghrelin [137]. It was also shown that ghrelin's influence on ocular hypertension is related to prostaglandins and nitric oxide production [137].

Recent studies show that ghrelin is produced by the ciliary processes and ghrelin receptor is also expressed in the trabecular meshwork and ciliary processes stroma. These data increase the already existing evidence for the ghrelin-GHSR system role in the ocular physiology. The endogenous production of ghrelin by the ciliary body together with the presence of the ghrelin receptor in the ocular tissue, namely, in the components responsible for aqueous humour dynamics, strengthens previous data that associated ghrelin with the pathophysiology of glaucoma. The hypotensive effect of ghrelin, its local production, and the GHSR-1a localization points to a possible therapeutic role in glaucoma but further studies are needed to clarify the mechanisms underlying ghrelin's influence in the ocular tension regulation.

### 3.13. Angiotensin II

It is already well known the role played by the renin-angiotensin system (RAS) in controlling systemic arterial pressure and ionic homeostasis. Angiotensin II (Ang II) is an endogenous potent vasoconstrictor that modulates systemic blood pressure by activating the G protein-coupled angiotensin II receptor, type I (AT1) [107]. Many of the RAS system components have been identified in human and animal eyes. Just like angiotensin I, angiotensin II and angiotensinogen are not able to cross the blood-brain barrier, and they also cannot cross the intact blood-retina barrier [138], creating a tissue-localized RAS system. RAS expression and secretory function have been shown in cultured human and rabbit ciliary body nonpigmented epithelia [139]. It was also proved that Ang II is able to induce cell proliferation in bovine TM cells and to increase the synthesis of collagen *in vitro* [140]. After angiotensin II administered intracamerally was shown to decrease uveo-scleral outflow, despite no changes in IOP, in rabbits [9], Wang et al. used olmesartan, an antagonist of AT1 receptors, topically in monkeys with laser-induced unilateral glaucoma and observed a dosage-dependent reduction of the IOP [141]. Topical olmesartan was already in early phase II glaucoma clinical trials when the study was terminated; even though it produced some IOP reductions, the efficacy was insufficient with no clear dose-response relationship. 

A recently discovered RAS component, angiotensin-converting enzyme 2 (ACE2), can degrade Ang I to Ang (1–9) and Ang II to Ang (1–7), which in turn acts oppositely to Ang II [142], since it is an active vasodilator and antiproliferative molecule. Ang (1–7) mainly acts through a new angiotensin receptor type, Mas receptor, which is exclusive of the angiotensin (1–7) ligand [13]. Vaajanen et al. [143] assessed the effect of angiotensin II and its angiotensin (1–7) metabolite in IOP levels and aqueous humor flow in rabbits with normal ocular tension. Angiotensin (1–7) reduced IOP, probably through the Mas receptors, without any changes in the aqueous humor release flow. Topical as well as intravitreal applications of angiotensin II led to a significant increased resistance to the drainage of aqueous humor. While this effect was reverted with an AT1 antagonist, Ang (1–7) had no effect. This suggests that Ang (1–7) reduces IOP via reduced AH formation [139]. 

Apart from this, also ACE1 inhibitors or AT2 receptor blockers have human studies with proven effect on IOP when administered orally. The authors described a reduction in both normotensive and glaucomatous persons' IOP, although blood pressure was lowered only in patients with arterial hypertension [139]. The mechanism proposed for the hypotensive effect of ACE1 inhibitors is the decrease in the production of AH by reducing blood flow in the ciliary body [144]. Additionally, ACE1 inhibitors also promote the synthesis of prostaglandins by preventing the breakdown of bradykinin, which could in turn lower IOP by increasing the uveoscleral outflow [139]. 

New antiglaucomatous drug therapies can be directed towards increasing ACE2 activity and consequently increasing the formation of Ang (1–7) or towards the activation of Mas receptors directly.

### 3.14. Serotonin

Serotonin (5-hydroxytryptamine, 5-HT) is a biogenic monoamine neurotransmitter, a result of the hydroxylation of tryptophan and decarboxylation of tryptophan hydroxylase [107]. Seven different receptor families have been identified (5-HT1 to 5-HT7).

The interest in the serotonin pathway for glaucoma therapy started with the report of elevated IOP or angle-closure glaucoma in patients under treatment with serotonin reuptake inhibitors (SSRIs) [145]. Since these drugs enhance serotoninergic neurotransmission, a possible role for serotonin antagonists arose. Opposite from what was thought, 5-HT2 agonists, but not 5-HT1A agonists or 5-HT2 antagonists, lowered IOP when applied topically in normotensive or cynomolgus monkeys' hypertensive eyes [107]. Studies with R-2,5-dimethoxy-4-iodoamphetamine, (R-DOI; a 5-HT2A,2B,2C receptor partial agonist) showed a decreased IOP in cynomolgus monkeys, mainly through an increased uveoscleral outflow, but also led to a small increase in AH formation [146]. A differential expression of the mRNAs of several receptor subtypes has been identified in human ocular tissue, with a high density of mRNAs for 5-HT2A and 5-HT2B in the TM, with much lower levels of 5-HT2C, 5-HT5, and 5-HT7 [147]. Interestingly, in the rabbit, topical application of 5-HT can either lower or elevate IOP. This is probably due to the presence of 5-HT receptors with opposing effects, such as 5-HT1 that reduces and 5-HT7 that elevates IOP [16]. A phase II clinical trial with BVT.28949, a 5-HT2A receptor antagonist, reduced IOP by 10% with a 4-week treatment, in patients with POAG or OH [148].

On the other hand, AL-34662, a 5-HT2 receptor agonist, was able to reduce IOP by 33%, with minimal local side effects, in cynomolgus monkeys [147]. Further studies are being undertaken regarding this compound.

Serotonin receptors have proven to be an effective target for glaucoma therapies, since they can lower IOP. It remains to be understood the true function of each of its receptors, since antagonic results can occur.

### 3.15. Forskolin

Forskolin is a flavonoid that can lower IOP in rabbits, monkeys, and humans, through an increase in cAMP (cyclic adenosine monophosphate) levels, when applied topically in the eye [15, 149].

It consists in a lipid-soluble compound that stimulates the enzyme adenylate cyclase, increasing cAMP levels, which, in turn, act upon the ciliary epithelium, reducing aqueous humor inflow [150]. Apart from topical usage, also oral forskolin has proven to be effective. Forskolin as a food supplement decreased IOP by 20% of the initial value, reversible upon suspension of the treatment [151]. Forskolin has a different molecular mechanism from any previously used antiglaucoma drug, which makes it promising for combination therapies.

## 4. Conclusion

No current medication for POAG is able to reduce the IOP by more than 25%–30%. Future strategies for treatment of glaucoma must therefore take into account different mechanisms to lower IOP. Better knowledge about trabecular outflow pathway, namely, mechanisms associated with phagocytosis, cytoskeletal reorganization, cell adhesion, and matrix deposition opened a new horizon for new hypotensive class agents. The main cause of elevated IOP in POAG is thought to be an increased outflow resistance through the pressure-dependent trabecular outflow system, so there is crescent interest in increasing TM outflow by extracellular matrix remodeling and/or by modulation of contractility/TM cytoskeleton disruption. There are also other agents that act by decreasing aqueous humour production or increasing uveoscleral outflow by different mechanisms from those drugs available for glaucoma treatment. We described some physiological targets that are being currently evaluated for their role in the IOP modulation. From those, some could generate a hypotensive drug with possible clinical use.

## Figures and Tables

**Table 1:** New therapeutics targets for IOP lowering and possible mechanisms of action.

Pathways	Mechanisms of action
Rho kinase	Modulation may increase TM outflow by modulation of contractility/TM cytoskeleton disruption
Endothelin-1	Modulation by may increase TM outflow by modulation of contractility/TM cytoskeleton disruption
Transforming growth factor-*β*	Modulation may increase TM outflow by remodeling extracellular matrix and/or by modulation of contractility/TM cytoskeleton disruption.
Connective Tissue Growth Factor	Modulation may increase TM outflow by remodeling extracellular matrix
Nitric Oxide	Modulation by may increase TM outflow by modulation of contractility TM cells
Angiopoietin-like molecules	Modulation may increase TM outflow by remodeling extracellular matrix
Adenosine	Modulation may increase TM outflow by remodeling extracellular matrix
Latrunculins	Modulation may increase TM outflow by modulation of contractility/TM cytoskeleton disruption
Cochlin	Modulation may increase TM outflow by potential mechanosensing mechanism
Cannabinoids	Modulation may decrease AH production, and/or may increase TM outflow (by increase in the dimensions of Schlemm's canal and/or by remodeling extracellular matrix), and/or may increase uveoscleral outflow
Melatonin	Modulation may increase TM outflow trough cholinergic and noradrenergic systems
Ghrelin	Modulation may increase TM outflow and/or uveoscleral outflow.
Angiotensin II	Modulation may increase uveoscleral outflow and/or decrease AH production
Serotonin	Modulation may increase uveoscleral outflow and decrease AH production
Forskolin	Modulation may decrease AH production
